# Sample and data random management in a medical laboratory equipped with automatic analysers

**DOI:** 10.1155/S146392468200042X

**Published:** 1982

**Authors:** G. Barbaresi, G. E. Martorana, C. Zuppi, A. Castelli

**Affiliations:** Istituto di Chimica Biologica Università Cattolica S. Cuore Facoltà di Medicina e Chirurgia Via della Pineta Sacchetti 664 Rome 00168 Italy


Journal of Automatic Chemistry, Volume 4, Number 4 (October–December 1982), pages 169–173

### Sample and data random management in a medical laboratory equipped with automatic analysers

G. Barbaresi, G. E. Martorana, C. Zuppi and A. Castelli

*Istituto di Chimica Biologica, Università Cattolica S. Cuore, Facoltà di Medicina e Chirurgia, Via della Pineta Sacchetti 664, 00168 Rome, Italy*

### Introduction

Recent advances in the performance of automatic analysers and in computer technology, although both share the aim of increasing speed, ease and operational capacity, have paradoxically led to a stalemate in many clinical laboratories. The new autoanalysers equipped with computers are capable of coping with a large analytical load, but often they can only partly cope with the data management process and so they increase laboratory tasks. Further, many hospital data processing centres (DPCs), copying their old manual procedures are limited to clerical work, which is a great pity considering their enormous computing powers [1].

Laboratories often find that their advanced instrument analytical capability is conditioned by two bottlenecks: manual input of test requests and memorization of clinical results [2]. Since a central data-bank is necessary for the complete clinical and statistical use of laboratory data in a large university hospital [3], a computerized system has been developed in the authors' clinical chemistry laboratory which is connected to the DPC by means of floppy disc information transfer. The system allows positive sample identification by an automatic optical reader and, together with on-line analytical result acquisition, it is possible to process patient specimens at random as they arrive in the laboratory. This feature means that the delays and difficulties encountered with an exclusively centralized management are avoided.

### Materials and methods

### *Hardware*

The DPC in the authors' teaching hospital is equipped with an IBM system, which is used for general accounting and, by means of terminals and printers, for the admission office and other centralized services and laboratories. The authors' laboratory uses the DPC as a data-bank (it memorizes patient requests and results, prints-out worksheets and clinical reports).

The central system (see figure 1) consists of the following instruments:

| | | |
|---|---|---|
| IBM | 370/138 | (CPU [1 Mbyte memory]). |
| IBM | 3340 | (data-bank). |
| IBM | 3344 | (disc unit). |
| IBM | 3741 | (recording unit). |
| IBM | 3287 | (printing terminal). |
| IBM | 2741 | (printing terminal with OCR-B characters). |
| IBM | 1403 | (printer). |
| IBM | 3747 | (disc-tape conversion unit). |

The laboratory system is based on a minicomputer (the P 6060:Ing. C. Olivetti & C. S.p.A., Ivrea, Italy), with a 32-Kbyte ROM and 400 Kbyte RAM in floppy discs, equipped with two interfaces for Olivetti standard peripherals and for analytical instruments. The system comprises the following:

| | | |
|---|---|---|
| Olivetti | 6602 | (CPU). |
| Olivetti | 2132 | (ROM). |
| Olivetti | 6600 | (IPSO standard Olivetti peripheral interface). |
| Olivetti | PR 1230 | (printer [130 characters/s]). |
| Olivetti | OPR 1830 wand | (automatic optical reader for OCR-B characters). |
| EIA | | (series interface for on-line 'modem' and/or 'current loop' connection with instruments). |
| SMAC | | (supplied by Technicon Inc., Tarrytown, New York, USA—a sequential multichannel analyser computerized for serum and plasma samples). |
| CLINICAB | | (supplied by the AMES Company, Division Miles Laboratory Inc., Elkhart, Indiana, USA, an automated urinalysis system). |

### *Software*

Programs for the P 6060 and connected peripherals are in BASIC and were written by laboratory staff. IBM programs mentioned in this paper were suggested by the authors and then written by DPC personnel.

### System description

#### *Sample flow*

When the patients' samples arrive in the laboratory, they are pretagged in OCR-B characters. These tags are automatically produced when a patient is admitted to the hospital and they are sent to the wards together with the clinical report. Samples for automatic instruments are easily recognizable: their container has a coloured cap; they are analysed quickly in a completely random manner. The results, sequentially furnished by the analyser on-line to the minicomputer, are associated with the sequence of the patient numbers printed on tags which are read by the OPR 1830 wand (see figure 2). In this way a 'result file' composed of many records is automatically generated; each record is made up of analytical data and of patient identification (ID) numbers.

0142-0453/82/0404 0169 $05·00 © 1982 Taylor & Francis Ltd

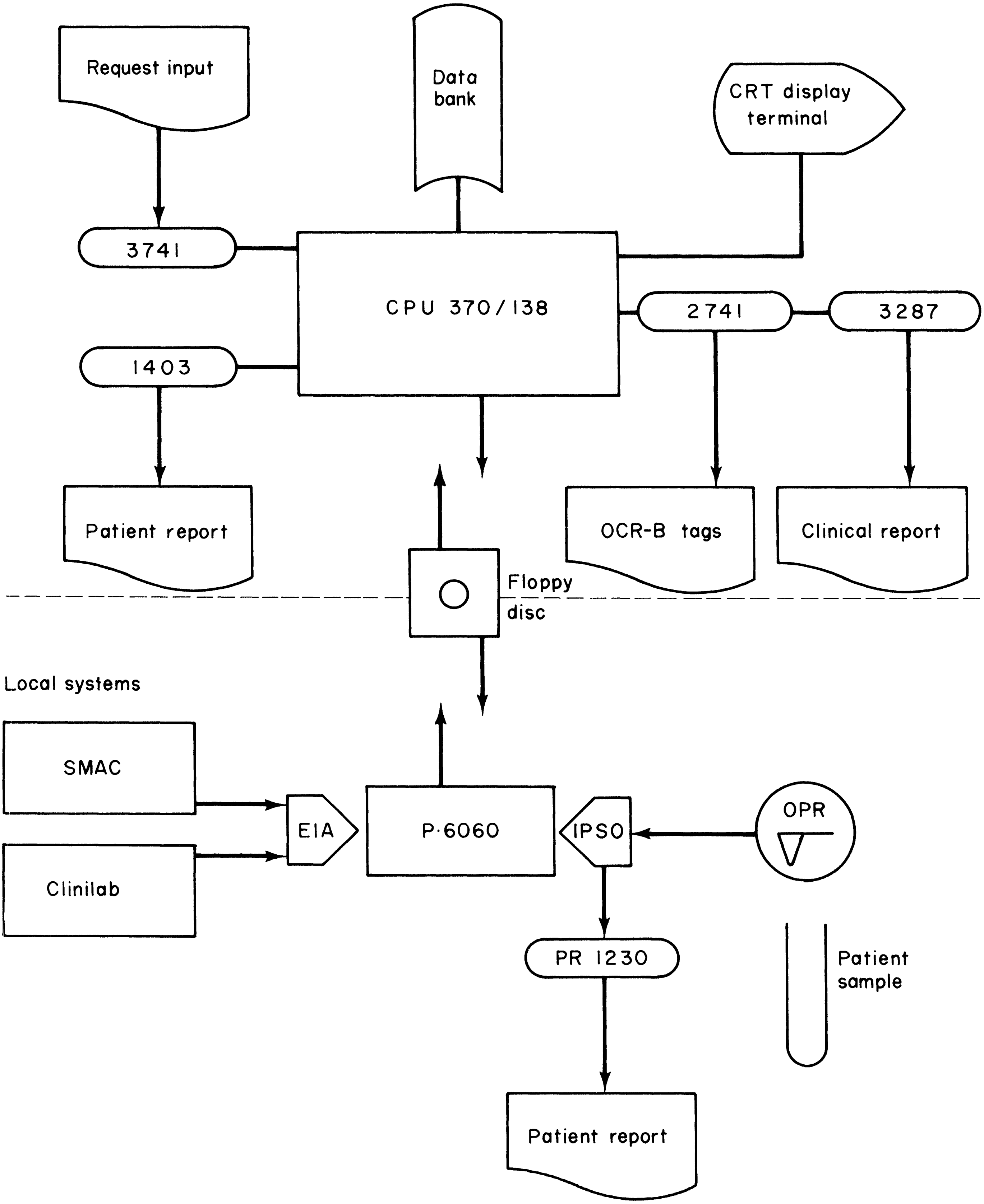

*Figure 1. Hardware configurations of the central and local systems.*

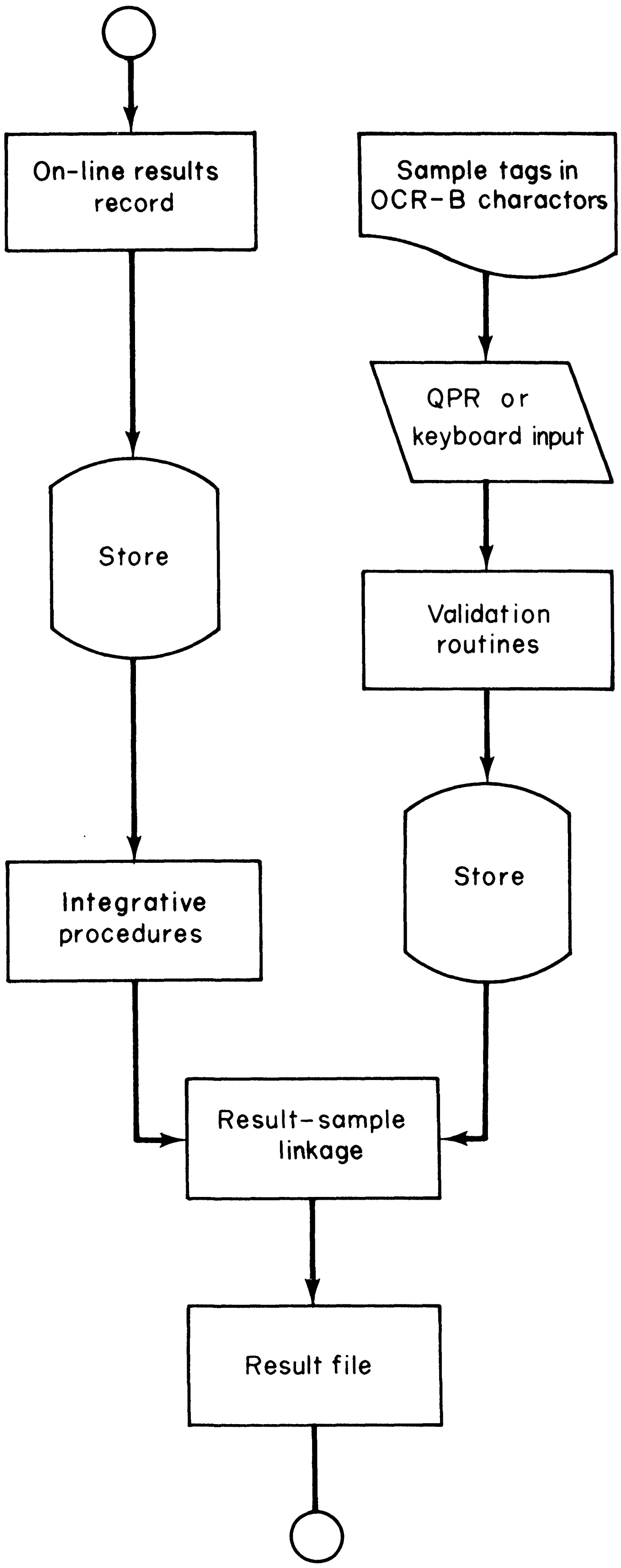

*Figure 2. Result-sample linkage.*

System efficiency depends upon the analytical and ID sequence, so a validation routine is also included. If automatic optical reading is not possible (as a result of blood spots, misprinting etc.), then manual input by keyboard, used as a remote peripheral, is allowed.

*Request flow*

Patient requests are drawn up in a cumulative list for each ward and sent directly to the DPC, where they are stored, processed, sequenced and, finally, printed on traditional worksheets, disregarding test codes which require automatic instrument analysis (see table 1). For these latter requests, a sequential file, made up of 128 byte records, is generated on floppy disc. The first 32 bytes of each record are reserved for the memorization of the patients' numbers (six bytes), of the ward codes (two bytes) and of the patients' names (24 bytes). The following 68 bytes are divided into 17 fields, four bytes long, which are flagged only when the corresponding test code is requested.

File data are also tabulated.

*Table 1. Analytical tests: code and field number.*

| *Test* | *Code* | *Field* |
|---|---|---|
| Glucose | 1 | 1 |
| Urea nitrogen | 2 | 2 |
| Total protein | 3 | 3 |
| Albumin | 5 | 4 |
| Sodium | 6 | 5 |
| Potassium | 7 | 6 |
| Calcium | 17 | 7 |
| Chloride | 20 | 8 |
| Phosphorus | 19 | 9 |
| Creatinine | 14 | 10 |
| Uric acid | 15 | 11 |
| Total bilirubin | 11 | 12 |
| Alkaline phosphatase | 10 | 13 |
| GOT | 8 | 14 |
| GPT | 9 | 15 |
| Cholesterol | 16 | 16 |
| Urinalysis | 29 | 17 |

### Result-request linkage (see figure 3)

*SMAC analytical results*

SMAC data records are logically set in numerical order by patient number and matched with request records; only requested results are stored. It is possible to manually enter those results which, because of momentary instrument failure, sample turbidity, out of range values and carry-over, for example, were not correctly supplied by the SMAC, or which were considered unreliable by a real-time quality control program [4].

At the end of the linkage procedure, patients' numbers without requests and their related results are automatically listed. Similarly, outstanding requests are printed out. Finally, pathological results of non-requested parameters are reported in a printed list.

*CLINILAB urinalysis results*

CLINILAB data records generate worksheets for sediment microscopy and for glucose and protein quantitative assays following analysed sample sequence (see figure 4). Up to five parameters for each sample are provided in the microscope examination worksheet. Each parameter is identified by a three-digit number: two for the kind and one for the quantity. In the work-list for glucose and protein quantitative assay only those samples with abnormal qualitative results are printed, each urine specimen being identified by the patient number and by the CLINILAB sequence number.

Analytical results can be entered by keyboard, by worksheet or both and input accuracy is controlled by a validation routine.

Urine chemistry and microscopy results are stored on a 37-byte record: a six-byte field for patient number, a two-byte field

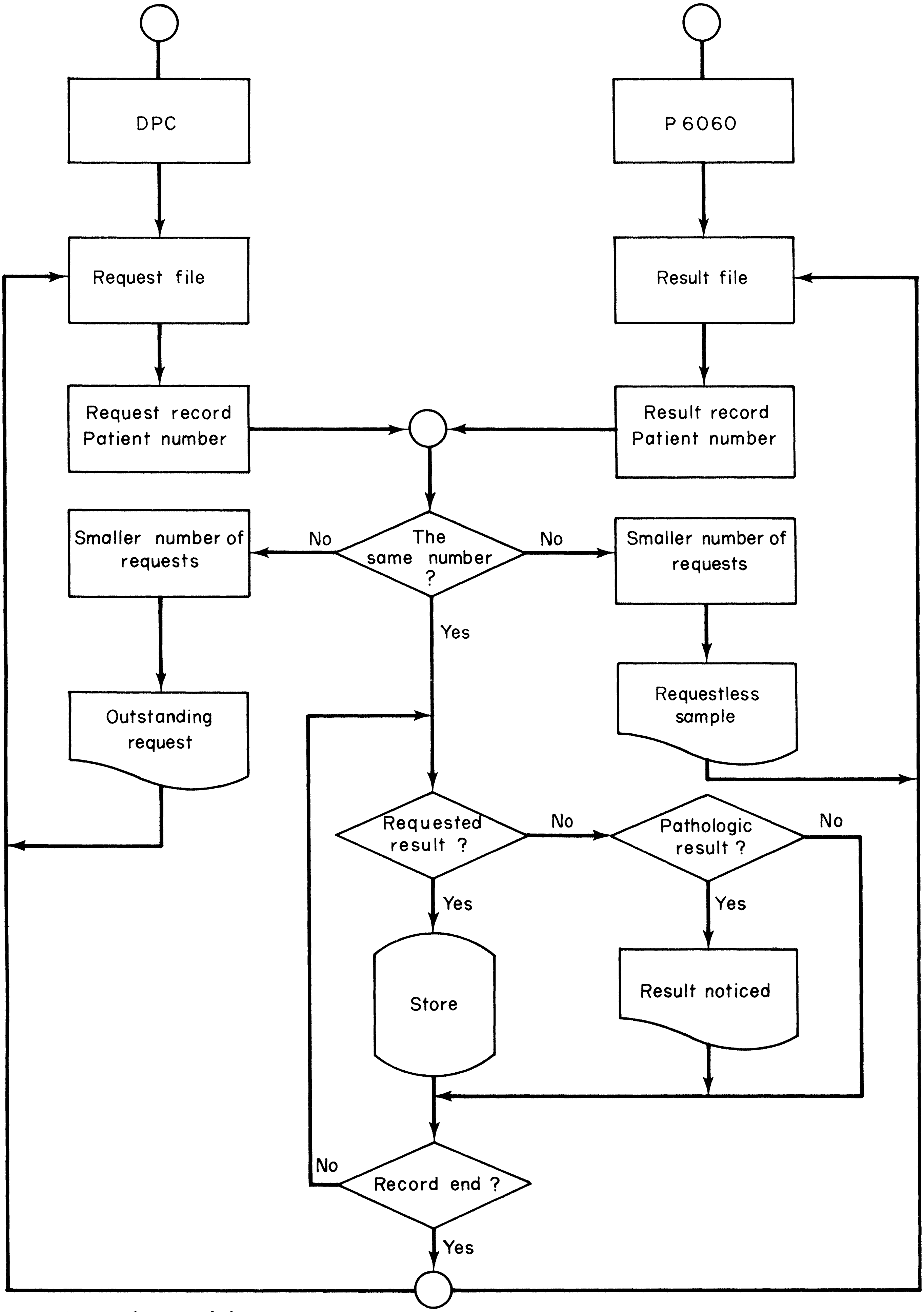

*Figure 3. Result-request linkage.*

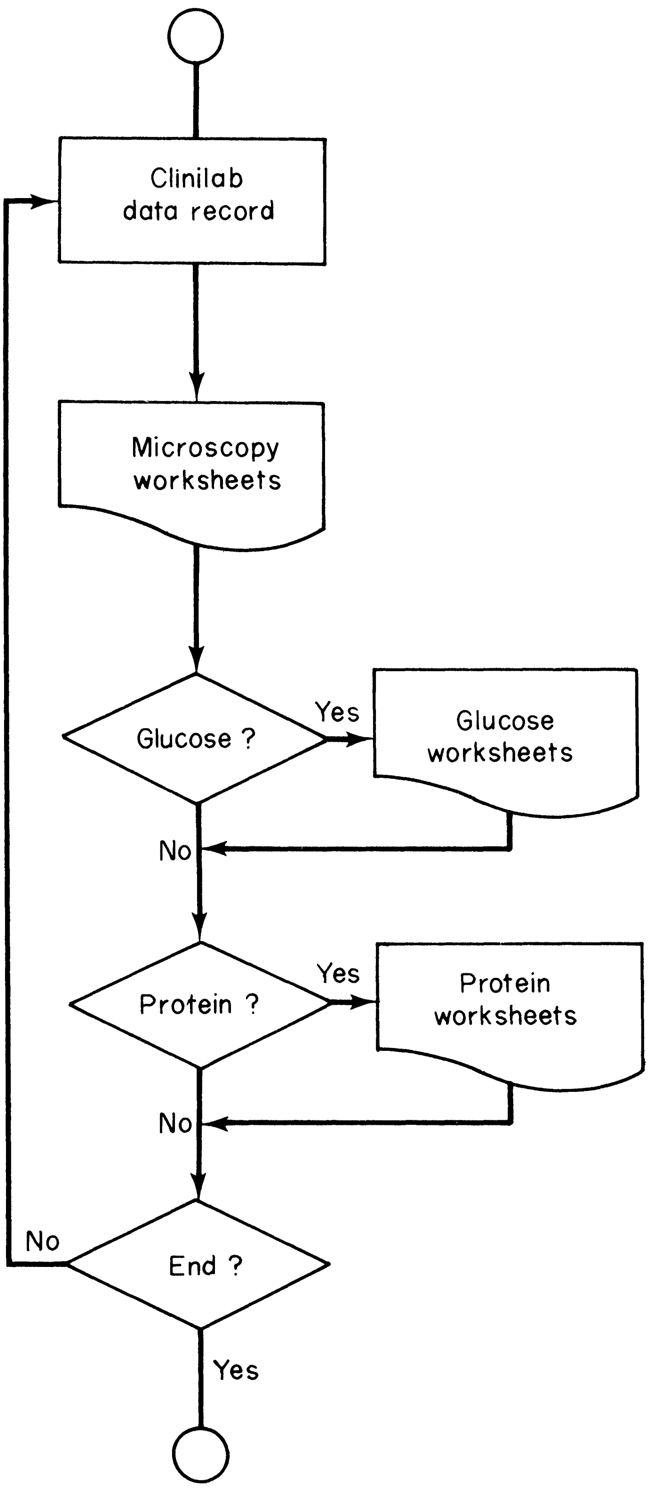

*Figure 4. Subsidiary worksheets generated from CLINILAB data records.*

for specific gravity and a two-byte field for pH; six fields, one byte each, for qualitative assays (glucose, protein, haemoglobin, ketone bodies, bilirubin and urobilinogen); two fields, three-bytes each, for possible glucose and protein quantative determinations and, finally, five three-byte fields for microscope findings. All these records are numbered in order and then matched with request records for linking. At the end of this procedure, results of patient samples without requests and outstanding requests are automatically noted.

*Report print-out*

The original request file is transformed into a result file after testing and is transferred to the DPC for processing and report printing. Reports can, if rèquired, be printed directly by the laboratory's PR 1230, ward by ward.

### Discussion

When the SMAC was introduced into the authors' laboratory in 1978, analytical capability was considerably increased and the laboratory's clerical work-load grew with it. Many manual operations had to be performed to personalize clinical reports and SMAC keyboard entry of census data takes up a lot of time. This work is also unnecessary because these data are already on file in the DPC. The authors also discovered that manual linking of the SMAC IDEE system with patient number and related data is liable to human error. Another drawback of the usual SMAC procedure is that its reports, printed on paper, cannot be directly transferred to a data-bank unless they have previously been transformed by further manual procedures.

As far as CLINILAB is concerned, the instrument gives a non-personalized report limited to physical and qualitative tests.

In order to fully exploit the SMAC's analytical capacity, which is more than 1500 tests, and to complete the CLINILAB urinalysis reports, a local information system was developed by the authors. It is integrated with the central system and permits the random management of samples and results and the automatic transfer of information to and from the DPC. The adoption of an automatic optical reader (an OPR 1830 wand) permits sample identification and, therefore, on-line acquisition of 'personalized' result records. With the use of a floppy disc, census and test request data are transferred from the DPC to the laboratory each day. The authors' program provides for automatic linking of information from the DPC and of the results from on-line analysers. These data, processed and completed if necessary, are stored on floppy disc. The authors prefer floppy disc to tape and to remote on-line connection because tapes do not permit a data output control and validation routine [5], and on-line connection would mean that the laboratory would be dependent on the DPC. With this type of distributed laboratory computing, the problems of analysers with digital output, but not provided with autonomous computerized data equipment, like CLINILAB, are also solved.

The use of floppy disc for data exchange between the laboratory and the DPC allows easy information retrieval for clinical and statistical research. The system proposed in this paper is cheap, has a proven versatility and is very easy to use.

### Acknowledgements

This work was supported by the CNR research grant N.80.02256.83, as part of the 'Preventive medicine' project.

### References

1. Whitcomb, C. C., Vogt, C. P. and Wilbur, N. M., *Computers in Biology and Medicine*, **8** (1978), 197.
2. Groves, W. E., *American Journal of Medical Technology*, **44** (1978), 575.
3. Wycoff, D. A. and Wagner, J. R., *American Journal of Clinical Pathology*, **70** (1978), 390.
4. Barbaresi, G., Martorana, G. E., Zuppi, C. and Castelli, A., *Computers in Biology and Medicine* (accepted for publication).
5. Goguel, A., *Nouvelle Revue Française d'Haematologie*, **20** (1978), 329.

